# Supplementary material for: Unmet Needs of Australians in Endometriosis Research: A Qualitative Study of Research Priorities, Drivers, and Barriers to Participation in People with Endometriosis
Source: Medicina (Kaunas). 2023 Sep 13;59(9):1655. doi: 10.3390/medicina59091655 (PMC10536628; doi:10.3390/medicina59091655)
Supplement: Supplementary file 1 [file medicina-59-01655-s001.zip › medicina-2556117-supplementary.pdf]

Focus Group Questions – Women with Endometriosis

1. How long have you been diagnosed with endometriosis?

2. What are the most bothersome symptoms for you that effect your:
    a. Personal and social life (including friendships and romantic relationships)
    b. At work or study
    c. Feelings about yourself or your body
    (probe for why these are the most important)

3. Are there certain aspects of your life that are most affected by your endometriosis? What are they ? and why are these the most significant for you?

4. Apart from pain, what are the most important things (like fatigue or sleep) that you think we should measure in a research study ?

5. What do you do to manage your pain and other symptoms? (if only pharmaceuticals are mentioned, prompt for lifestyle and other interventions)
    (followup questions)
    a. Were any of these beneficial for you ? what symptoms did you notice a change in?
    b. Were there any you felt made you feel worse ?

6. What areas of endometriosis do you think need more investigation/funding or time spent on them? Why are these important to you ? (probe towards management options rather than just a cure)

7. Research projects often ask people to do something (such as yoga) or take something (like a medicine) This all takes time. If you were part of a research project, how much time per week would be realistic to spend on:

    a. *Doing* something,, if this required some kind of physical involvement (like a yoga class). Would it be any different if you had to do this at home rather than going somewhere ?
    b. *Taking* something, like a pill or a powder

8. Opiods (like Endone) are commonly used by women with endometriosis due to severe pain. If we were investigating another form of pain reduction (such as by using medicinal cannabis), would this interest you ? why ?

9. The other part of research projects are measuring changes. This might be things like changes in your pain, or changes in your blood. What would be realistic for you in terms of how often we should measure things and how we should do it ?
    a. How would you feel about completing most of your diaries or other measurement tools online rather than on paper ?
    b. Blood tests are often important for safety, especially if you are taking something. Are there any things we could do to make this easier for you? Would having to have blood tests every 4-6 weeks stop you from joining a study?

10. What would be the biggest barrier(s) to being part of a research project on endometriosis for you?

11. Would financial incentives be important in making a decision to participate in a study (e.g gift cards for any travel time) ? why ? why not?

12. Do you currently look for research studies to be part of ? where do you look ? where/how do you think we should tell people about our upcoming studies that they might want to join?

13. Sometimes getting enough research funding for endometriosis is difficult. Some researchers have suggested crowdfunding might be a possible option. Have you heard of crowdfunding? Have you ever been part of crowdfunding before ? Would you crowdfund research ? why, why not ?

14. Anything else you would like to tell us?
